# Cohort studies of cardiovascular disease in the Seychelles, Tanzania and Mauritius

**Published:** 2012-05

**Authors:** Pascal Bovet, Conrad Shamlaye

**Affiliations:** University Institute of Social and Preventive Medicine, Lausanne, Switzerland; Ministry of Health, Republic of Seychelles

We read with interest the review by Kengne *et al.* on cohort studies of cardiovascular disease in sub-Sahara Africa.^1^ We agree with the authors that cohort studies are important tools to advance our knowledge of cardiovascular disease in the region and inform appropriate clinical and public health responses.

We recognise the difficult challenge of identifying all cohort studies in the region. We wish however to mention several cohort studies in the Seychelles, Tanzania and Mauritius, which were published in leading medical journals but were not included in the review, although they met inclusion criteria set by the authors of the review.

The Republic of Seychelles, which lies in the Indian Ocean around 1 000 km east of Kenya, belongs to south Saharan Africa. Seychelles is part of WHO AFRO, is a member of the South African Development Community (SADEC) and contributes epidemiological data to the Global Burden of Disease project for estimates of the east Africa region. The majority of the population of Seychelles is of African descent.

In a cohort study of 5 514 Seychelles children, there was a strong association between weight gain during the first year of life and overweight/obesity at age five to 17 years.^2^ Adherence to antihypertensive treatment was low in 50 hypertensive patients followed for 12 months, despite free healthcare.^3^ In this study adherence was measured with electronic pill containers, the gold standard for assessment of therapeutic adherence. In a cohort study of 153 smokers followed for six months, smokers who were shown pictures of their own atherosclerotic plaques in their carotid arteries (B-mode ultrasonography) had improved rates of smoking cessation.^4^ A cohort study among 644 Seychelles children enrolled at birth showed no overall effect of pre-natal exposure to organic mercury on blood pressure (BP) levels at age 12 and 15 years.^5^

In Tanzania, 653 participants with BP ≥ 160/95 mmHg and 653 with BP < 160/95 mmHg from a population survey of 9 254 subjects in Dar es Salaam had BP readings on three additional visits over an eight-week follow-up period. Their BP decreased markedly over subsequent visits, irrespective of baseline BP levels, and the prevalence of hypertension dropped by approximately 50% based on BP values on the second, third or fourth visits, compared to BP values on the first visit.^6^

In a subsequent 12-month cohort study, 161 untreated Tanzanian participants who had BP ≥ 160/95 mmHg on four separate visits were advised to seek healthcare. Twelve months later, only 34% reported to have attended a healthcare provider and antihypertensive treatment was taken by only 34% at some point during and 3% at the end of the 12-month follow up.^7^

Mauritius is also part of sub-Saharan Africa although a substantial proportion of the population is of Indian descent. Many large cohort studies have been performed there. We mention just two,^8^,^9^ as it is not possible to include all of them in the context of this letter.
